# Assessment of a New Gait Asymmetry Index in Patients After Unilateral Total Hip Arthroplasty

**DOI:** 10.3390/jcm13226677

**Published:** 2024-11-07

**Authors:** Jarosław Kabaciński, Lechosław B. Dworak, Michał Murawa

**Affiliations:** 1 Department of Biomechanics, Poznan University of Physical Education, Krolowej Jadwigi 27/39, 61-871 Poznan, Poland; murawa@awf.poznan.pl; 2 Faculty of Medicine and Health Sciences, The President Stanislaw Wojciechowski Calisia University, Nowy Świat 4, 62-800 Kalisz, Poland

**Keywords:** total hip arthroplasty, pathological gait, asymmetry index, biomechanics

## Abstract

**Background/Objectives:** Comparing a given variable between the lower extremities (LEs) usually involves calculating the value of a selected asymmetry index. The aim of this study was to evaluate the mean-dependent asymmetry index for gait variables. **Methods:** The three-point crutch gait asymmetry between the non-surgical LE (NS) and surgical LE (S) was assessed in 14 patients after unilateral total hip arthroplasty. An eight-camera motion capture system integrated with two force platforms was used. The values of the new gait asymmetry index (MA) were calculated for such variables as stance phase time (ST), knee flexion and extension range of motion (KFE RoM), hip flexion and extension range of motion (HFE RoM), and vertical ground reaction force (VGRF). **Results:** An analysis related to gait asymmetry showed significantly higher values for all variables for the NS than for the S (the MA ranged from 9.9 to 42.0%; *p* < 0.001). In the case of comparisons between the MA and other indices, the intraclass correlation coefficient ranged from 0.566 to 0.998 (*p* < 0.001) with Bland–Altman bias values that ranged from −18.2 to 0.3 %GC (ST), from 0.0 to 0.5° (KFE RoM), from −12.4 to 1.4° (HFE RoM), and from −11.9 to −0.1 %BW (VGRF). **Conclusions:** The findings revealed a prominent three-point crutch gait asymmetry for all variables, especially a disturbingly large asymmetry for the HFE RoM and VGRF. The comparisons also showed generally excellent or good agreement with the other indices. Furthermore, the mean MA result from *n* single values was the same as the MA result calculated using the mean values of a given variable. The MA, as an accurate asymmetry index, can be used to objectively assess pathological gait asymmetry.

## 1. Introduction

Human gait analysis commonly involves comparisons of selected variables between one lower extremity (LE) and the opposite LE. Thus, the asymmetry of pathological gaits in patients with various dysfunctions and normal gait in healthy individuals are assessed [1,2,3,4]. The percentage result of a comparison between both LEs is provided by the gait asymmetry index (AI) calculated for a given variable such as the spatiotemporal parameter, angle, joint moment, or ground reaction force [2,4,5,6].

In particular, AI values are disturbingly high for pathological gaits, often exceeding five percent or more [2,4,7]. Too much gait asymmetry can lead to increased energy costs, balance disorders, and abnormal joint loading [4,8]. Therefore, a low AI, indicating a non-significant deficit in the value of a given variable for the surgical LE compared to the non-surgical LE, is recommended in the pursuit of a human gait pattern because almost symmetrical movements are a sign of a biomechanically correct gait [9,10].

Assessments of gait asymmetry are provided by indices such as the standard ratio index [11], the ratio of the difference for a given variable between the LEs and their arithmetic mean [1], and Vagenas and Hoshizaki’s formula [12] as well as the normalized symmetry index containing the minimum and maximum values from *n* trials [6]. The AI value can also be determined using the natural logarithm [13] and the symmetry angle [14]. In turn, for the so-called symmetry function in the gait cycle, the percentage difference in the value of a given variable between LEs in relation to their range is calculated as a function of time [7,9,15]. In addition, other studies have defined the global gait asymmetry index [16], the symmetry index for discrete variables [17], and the relative asymmetry index [18].

In the rehabilitation process of patients after total hip arthroplasty (THA), there is also a clinical need for objective measurements of biomechanical gait parameters to assess functional progress [19,20,21,22]. Although THA reduces pain [23,24,25,26] and improves mobility in these individuals [21,25,26], gait abnormalities may remain. Hence, significant importance is attributed to the assessment of gait asymmetry in terms of spatiotemporal, kinematic, and kinetic variables [24,27,28,29,30,31] as well as to the comparison of gait biomechanics between the preoperative and postoperative time points [21,32,33,34] and between different surgical approaches [20,32,35,36]. Furthermore, the gait asymmetry index results in a given patient are important in the context of assessing the effects of his rehabilitation until the restoration of a normal gait pattern. An analysis of the available studies indicated insufficient data on the asymmetry of the three-point crutch gait for biomechanical parameters in patients after THA. In addition, previous gait asymmetry indices were not based on normalization to 100% of the mean value of a given variable from *n* trials for the non-surgical LE. In this study, it was assumed that the new index would meet this condition and that the mean result from *n* single values would be equal to the result when this index was calculated using the mean values of a given variable. Considering these important aspects, this study aimed to evaluate the mean-dependent asymmetry index for gait variables in patients after unilateral THA.

## 2. Materials and Methods

### 2.1. Participants

Fourteen female patients (age: 58.5 ± 6.7 years, body mass: 76.4 ± 12.9 kg, and height: 1.65 ± 0.08 m) participated in this study. These women underwent unilateral total hip arthroplasty (THA) with a posterior surgical approach. The time from the surgical procedure to laboratory testing was 24 ± 3 days (in the range of 19–30 days). The patients were recruited from the orthopedic units of the hospitals. The participants fulfilled the following inclusion criteria: (1) age between 50 and 70 years, (2) only underwent THA for one lower extremity (LE), (3) use of a three-point crutch gait, and (4) no history of coexisting diseases that could influence the gait pattern. In the patients, the surgical LE (S) related to the THA and the non-surgical LE (NS) were defined.

The participants were acquainted with the experimental procedures, and they provided written informed consent. This study was approved by the Bioethical Committee at Poznan University of Medical Sciences (number 208/17) in accordance with the Declaration of Helsinki.

### 2.2. Data Collection

This study used an 8-camera motion capture system operating at a frequency of 200 Hz (Smart-D, BTS Bioengineering, Milan, Italy) synchronized with two stationary force platforms operating at 800 Hz (BP400600, AMTI, Watertown, MA, USA). According to the Vaughan–Davis model [37], nineteen special spherical markers (diameters of 20 mm) that reflected infrared rays were placed on the sacrum between the posterior superior iliac spines as well as between the right and left anterior superior iliac spines, on the femoral greater trochanter, on the bar on the lateral side of the thigh, on the femoral lateral epicondyle, on the head of the fibula, on the bar on the lateral side of the LE, on the lateral malleolus, on the calcaneus, and on the head of the fifth metatarsal [37,38]. The markers were tracked using BTS Smart Tracker software (version 1.1). The same researcher stuck the markers on each participant. Spatiotemporal, kinematic, and dynamic gait parameters were collected using BTS Smart Capture software. Moreover, data analysis was performed using BTS Smart Analyzer software.

The participants moved using the three-point crutch gait, which is often recommended for patients during rehabilitation after THA [39]. It provides varying levels of weight bearing [40]. In this pattern, both crutches are advanced simultaneously with the S to a frontal stance (three points), and the NS is then moved to it after a rear swing [39,40,41]. All participants walked at a self-optimal velocity for a distance of approximately 8 m on a measurement path. After several preparatory trials, each patient performed at least three successful walks for the S and NS independently in contact with one force platform (for a total of at least six test walks). Thus, the participant (1) rolled only the foot of the S on the force platform during the trials for the S (with the crutches and the NS on the ground) and (2) rolled only the foot of the NS on the platform during the trials for the NS (with the crutches and the S on the ground).

### 2.3. Data Analysis

The selected gait variables included (1) the stance phase time (ST) (spatiotemporal), (2) the knee flexion and extension range of motion (KFE RoM) (kinematics), (3) the hip flexion and extension range of motion (HFE RoM) (kinematics), and (4) the peak vertical ground reaction force (VGRF) (dynamics). The ST values were normalized to 100% of the gait cycle, whereas the VGRF values were normalized to the participants’ body weights (%BW). The results of these variables for 42 trials (14 participants × 3 trials) were submitted to further analysis.

In this study, a new gait AI (MA) was defined:

(1) $$\mathrm{MA}=\frac{X_{\mathrm{NS}}-X_{S}}{<X_{\mathrm{NS}}>}\cdot 100\%$$

where X_NS_ and X_S_ are the values of a given variable for the NS and S, respectively, related to a single trial, and <X_NS_> is the mean value of a given variable from *n* trials for the NS. Furthermore, <X_NS_> is always normalized to 100%. After replacing the NS with the D (dominant LE) and the S with the ND (non-dominant LE), formula 1 can be used to assess normal gait asymmetry.

In addition, other asymmetry and symmetry indices were used for comparison, i.e., the ratio index (RI) [11], the gait asymmetry index (GA) [12], the symmetry index (SI) [1], and the normalized asymmetry index (NSI) [6]. Formulas related to the RI, GA, SI, and NSI are presented in a study by Quinn et al. [6]. The values of these indices were also calculated using the mean results of the four analyzed variables (<MA>, <RI>, <GA>, and <SI>). Thus, apart from the NSI, this formula made it impossible to calculate mean results due to the denominator containing the minimum and maximum values of a given variable for *n* trials.

### 2.4. Statistical Analysis

The distribution of the data was verified via the Shapiro–Wilk test. The paired-samples t-test and the Wilcoxon test were used for comparisons between LEs. The independent t-test and the Mann–Whitney U test were used for comparisons between the indices. Effect sizes were calculated, and according to the Cohen’s *d* guidelines, the values were determined to be small (0.2), medium (0.5), or large (0.8) [42]. To analyze the absolute agreement between the indices, an intraclass correlation coefficient (ICC) with a 95% confidence interval (CI) related to the 2-way random single measures (2.1) was used. Moreover, Bland–Altman plots showing systematic bias and the limit of agreement (LOA) were created [43]. The ICCs were interpreted based on the following scale: <0.5 (poor), 0.5–0.75 (moderate), 0.75–0.9 (good), and >0.9 (excellent) [44]. The significance level (alpha) was defined as *p* < 0.5. Statistical analysis was conducted using IBM SPSS Statistics software for Windows, version 29.0 (Armonk, NY, USA: IBM Corp.). To calculate the AI, Microsoft Excel 2016 was used.

## 3. Results

The means and standard deviations of the ST, KFE RoM, HFE RoM, and VGRF are presented in Table 1. For all these variables, pairwise comparisons revealed significantly higher mean values for the NS than for the S in the patients (*p* < 0.001). The values of Cohen’s *d* for the comparisons between the LEs ranged from 1.0 to 3.7 (a very large effect size).

Table 2 and Table 3 show the results for the asymmetry and symmetry indices. The analysis demonstrated significantly lower values (1) for the MA than for the NSI (*p* < 0.001) for ST, (2) for the MA than for the NSI (*p* < 0.007) for KFE RoM, (3) for the MA than for the GA (*p* < 0.012) and the SI (*p* < 0.019) for HFE RoM, and (4) for the MA than for the GA (*p* < 0.004) and the SI (*p* < 0.006) for VGRF. The values of Cohen’s *d* for the comparisons between the AIs ranged from 0.0 (a very small effect size) to 0.9 (a large effect size).

**Figure 1:**
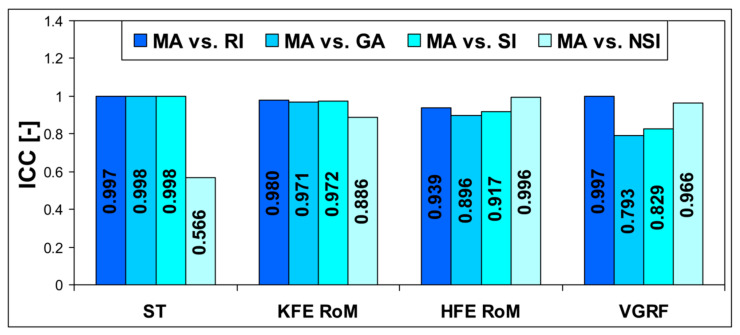
The ICCs for the absolute agreement between the MA and the RI, GA, SI, and NSI. ICC—the intraclass correlation coefficient, MA—the new asymmetry index, RI—the ratio index, GA—the gait asymmetry index, SI—the symmetry index, NSI—the normalized symmetry index, ST—stance phase time, KFE RoM—knee flexion and extension range of motion, HFE RoM—hip flexion and extension range of motion, VGRF—vertical ground reaction force.

The ICC results for the AIs are presented in Figure 1. The ICCs indicated excellent agreement between the MA and the RI, GA, SI, and NSI for all variables with a 95% CI = −0.074–0.999 (*p* < 0.001); good agreement between the MA and the NSI for KFE RoM with a 95% CI = −0.098–0.971 (*p* < 0.001); good agreement between the MA and the GA for HFE RoM with a 95% CI = −0.074–0.980 (*p* < 0.001); good agreement between the MA and the GA and SI for VGRF with a 95% CI = −0.113–0.950 (*p* < 0.001); and moderate agreement between the MA and the NSI for ST with a 95% CI = −0.200–0.827 (*p* < 0.001).

**Figure 2:**
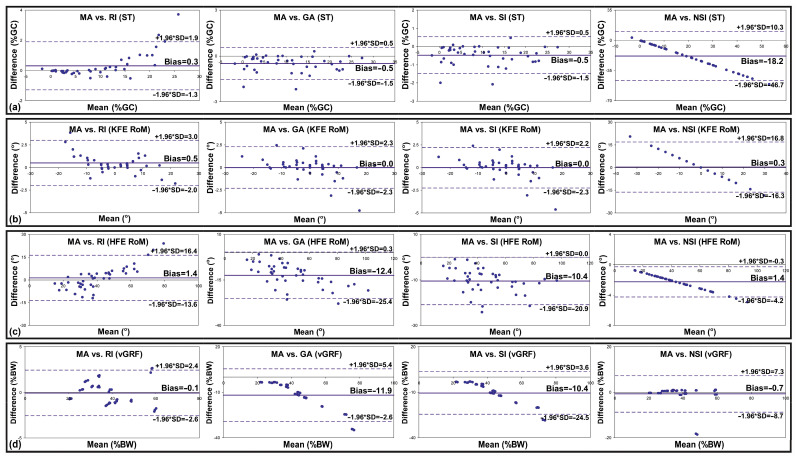
Bland–Altman plots for the MA vs. the RI, the MA vs. the GA, the MA vs. the SI, and the MA vs. the NSI for (**a**) ST, (**b**) KFE RoM, (**c**) HFE RoM, and (**d**) VGRF. MA—the new asymmetry index, RI—the ratio index, GA—the gait asymmetry index, SI—the symmetry index, NSI—the normalized symmetry index, ST—stance phase time, KFE RoM—knee flexion and extension range of motion, HFE RoM—hip flexion and extension range of motion, VGRF—vertical ground reaction force, SD—the standard deviation for the average difference between the two indices, +1.96 * SD—the upper limit of agreement, −1.96 * SD—the lower limit of agreement, bias—the average difference between the two indices.

The Bland–Altman plots (Figure 2) show a systematic bias as the mean difference between the MA and the RI, the MA and the GA, the MA and the SI, and the MA and the NSI in the ranges of −18.2–0.3 %GC (ST), 0.0–0.5° (KFE RoM), −12.4–1.4° (HFE RoM), and −11.9–−0.1 %BW (VGRF).

## 4. Discussion

The present study aimed to assess the mean-dependent asymmetry index for gait variables in patients after unilateral THA. The MA (formula 1) was used for the analysis. In the numerator, it contains the difference in the values of a variable between the NS and S. In the denominator, it contains the mean value of this variable from *n* trials for the NS. For calculations using this new AI, values of selected variables of the three-point crutch gait were used, such as ST, KFE RoM, HFE RoM, and VGRF.

The MA results for all variables indicated significant gait asymmetry in patients after THA (in the range of 9.9–42.0%), similar to what previous studies have shown in participants after THA for VGRF [3,39] and hip and pelvic angles [45]. In this study, the patients were examined shortly after leaving the clinic (approximately 24 days after surgery), and they still limped during the gait. Due to pain, weakness in the surgical hip joint, and a tendency to be less physically active, patients undergoing THA often demonstrate a pattern of prominent limping [46,47,48]. Furthermore, gait asymmetry is also a result of habits and compensatory mechanisms that already existed before implantation of the endoprosthesis [39,49]. In the case of the three-point crutch gait, the center of gravity of the patient shifts towards the NS side after THA, causing a greater load on the crutches on the NS side compared to the S side [39]. The limping of patients after surgery and the associated asymmetric loading of the NS may lead to overloading and degenerative disease of the non-surgery hip joint [3,47,50,51]. Hence, it is useful to conduct follow-up examinations of patients’ gait biomechanics during rehabilitation using a force platform and a motion capture system to objectively assess the degree of asymmetry. Undoubtedly, unfavorably large asymmetries should be reduced by effective rehabilitation and proper education of patients with the goal of obtaining a normal gait pattern because almost symmetrical movements mean a biomechanically correct gait.

Gait analysis commonly involves assessing the asymmetry of a given variable by comparing the S to the NS (pathological gait) or the ND to the D (normal gait). Higher results are often observed for the NS than for the S (as well as for the D than for the ND); thus, the formula should contain the difference in the values of a given variable between the NS and S (or the D and ND) multiplied by 100%. This condition is met by the MA; i.e., its numerator is this percentage difference. The ratio of (X_NS_ − X_S_)·100% and <X_NS_> (Formula (1)) allows the gait parameter deficit for the S relative to the NS to be determined.

During analysis, e.g., in Microsoft Excel, first a given index (asymmetry or symmetry) is used for a single trial. Then, the arithmetic mean (± the standard deviation) from *n* values of this index is calculated. Usually, the mean value of an index is different from the value of that index calculated using the mean results of a given variable for both LEs, although they should be the same. This irregularity is eliminated by the new AI, i.e., the MA, which differs from the RI, GA, SI, and NSI due to its denominator, which includes the arithmetic mean of all results of a variable (*n* trials) for the NS. According to this approach, the asymmetry results of the MA and <MA> are equal, in contrast to the RI, GA, SI, and NSI, whose values are different compared to those calculated using the arithmetic means of the analyzed variables, i.e., <RI>, <GA>, <SI>, and <NSI>.

For comparison, the values of other gait asymmetry (or symmetry) indices were also calculated. Apart from the GA, which is related to the natural logarithm, the remaining indices, i.e., the MA, RI, SI, and NSI, contain the difference between the NS and S in their numerators, resulting from the normalization of the value of a given variable for the NS to 100%. In most cases, the analysis showed similar results between the MA and the other indices. Importantly, the ICC results indicated excellent or good absolute agreement between the MA and the RI, GA, SI, and NSI (except for between the MA and the NSI for ST). Furthermore, this high agreement was confirmed by generally small Bland–Altman bias values for 15 of 16 plots (including 11 plots close to zero). Increased bias was only observed between the MA and the NSI for ST, resulting from the large dispersion of the NSI values.

Considering time graphs of the forces or angles in a joint, it is possible to determine the so-called gait symmetry function (SF). Some authors, using the SF formula related to the GA, presented graphs as a function of time for this index [7,9,18]. However, researchers commonly focus on the analysis of AIs calculated for extreme values and the RoM of selected gait parameters. Hence, the SF was not included in this study, although the MA can also be calculated for each value in a graph of a given variable over the gait cycle.

This study had limitations. First, unexpected difficulties with access to men meant that only women were included in this study. Secondly, the adopted inclusion criteria prevented the recruitment of a larger number of patients. Thirdly, a second measurement session was not conducted due to the refusal of some participants.

## 5. Conclusions

This study revealed significant gait asymmetry in patients after unilateral total hip arthroplasty for all analyzed variables. In particular, disturbingly large gait asymmetries for the hip flexion and extension range of motion (42.0%) and vertical ground reaction force (38.4%) were observed. Despite observing the patients in an early stage after the surgical procedure (3–4 weeks), smaller differences in the values of both variables were expected between the non-surgical and surgical lower extremities. Comparisons also showed generally excellent or good agreement between the MA, i.e., the new asymmetry index, and the other four indices. The MA met the important condition of equality between its mean result from *n* single values and the result calculated using the mean values of a given variable. Undoubtedly, the MA, as an accurate asymmetry index, can be used to objectively assess pathological gait asymmetry.

## Figures and Tables

**Table 1:** The means and standard deviations of the selected gait variables in the patients.

Variable	Surgical	Non-Surgical	*p*
ST (%GC)	60.4 ± 2.9	67.0 ± 3.5	<0.001 *
KFE RoM (°)	42.2 ± 6.5	48.8 ± 6.7	<0.001 *
HFE RoM (°)	22.4 ± 4.0	38.6 ± 6.5	<0.001 *
VGRF (%BW)	66.5 ± 12.5	108.0 ± 3.4	<0.001 *

Notes: ST—stance phase time, KFE RoM—knee flexion and extension range of motion, HFE RoM—hip flexion and extension range of motion, VGRF—vertical ground reaction force, GC—gait cycle, BW—body weight, *—significant difference between the surgical and non-surgical lower extremities.

**Table 2:** The means and standard deviations of the symmetry and asymmetry indices for the selected gait variables in the patients.

Variable	MA (%)	RI (%)	GA (%)	SI (%)	NSI (%)
ST	9.9 ± 7.8	9.5 ± 7.3	10.3 ± 8.2	10.3 ± 8.1	28.1 ± 22.4 *
KFE RoM	13.5 ± 7.8	13.4 ± 7.4	14.8 ± 8.7	14.7 ± 8.6	19.3 ± 11.1 *
HFE RoM	42.0 ± 18.9	40.4 ± 12.7	54.2 ± 22.9 *	52.3 ± 20.7 *	44.1 ± 19.9
VGRF	38.4 ± 10.9	38.5 ± 11.1	50.4 ± 9.5 *	48.9 ± 17.8 *	39.2 ± 11.6

Notes: MA—the new asymmetry index, RI—the ratio index, GA—the gait asymmetry index, SI—the symmetry index, NSI—the normalized symmetry index, ST—stance phase time, KFE RoM—knee flexion and extension range of motion, HFE RoM—hip flexion and extension range of motion, VGRF—vertical ground reaction force, *—significant difference between the indices.

**Table 3:** The results of four indices calculated using the mean values of the selected gait variables from *n* trials of each lower extremity in the patients.

Variable	<MA> (%)	<RI> (%)	<GA> (%)	<SI> (%)
ST	9.9	9.9	10.4	10.4
KFE RoM	13.5	13.5	14.5	14.5
HFE RoM	42.0	42.0	54.4	53.1
VGRF	38.4	38.4	48.5	47.6

Notes: <MA>, <RI>, <GA>, and <SI>—the new asymmetry index, the ratio index, the gait asymmetry index, and the symmetry index calculated using the mean results of a given variable, respectively; ST—stance phase time; KFE RoM—knee flexion and extension range of motion; HFE RoM—hip flexion and extension range of motion; VGRF—vertical ground reaction force.

## Data Availability

The data are available upon request from the corresponding author.
